# Bilateral multifocal Warthin's tumors in upper neck lymph nodes. report of a case and brief review of the literature

**DOI:** 10.1186/1746-160X-8-11

**Published:** 2012-04-03

**Authors:** Christian Naujoks, Christoph Sproll, Daman Deep Singh, Sebastian Heikaus, Rita Depprich, Norbert R Kübler, Jörg Handschel

**Affiliations:** 1 Department for Cranio- and Maxillofacial Surgery, Heinrich-Heine-Universität, Moorenstr. 5, D-40225 Düsseldorf, Germany; 2 Department of Pathology, Heinrich-Heine-Universität, Moorenstr. 5, D-40225 Düsseldorf, Germany; 3 Department for Cranio- and Maxillofacial Surgery, Heinrich-Heine-Universität, Moorenstr. 5 (Gebäude 18.73), D-40225 Düsseldorf, Germany

**Keywords:** Warthin's tumor, Bilateral, Multifocal, Neck lymph nodes, Treatment, Pathogenesis

## Abstract

Cystadenolymphomas (Warthin's tumors) are the second most frequent lesions of the parotid gland. Due to their benign clinical behavior, the low rates of recurrence and malignant transformation they were classified as tumor-like lesions. In addition, a polyclonal growth of the epithelial components of the tumor could be detected. Warthin's tumors occur bilateral in 7-10%, whereas a multifocal appearance is extremely rare. Even if the pathogenesis is still unclear a heterotopia of salivary tissue during embryogenesis is the most likely explanation for the origin of these tumors in the upper neck and periparotideal region. Here we present a rare case of bilateral, multifocal, extraglandular Warthin's tumors in lymph nodes of the upper neck and give a brief review of the literature. If a primary malignancy can be excluded by a careful staging procedure prior to the operation an isolated excision of the lesions of the neck is the adequate treatment.

## Introduction

Cystadenolymphoma is the second most frequent benign tumor of the parotid gland representing 6 to 10% of all tumors of the salivary glands [1]. In general, Warthin's tumors present as encapsulated lesions with cystic and solid areas which usually occur in the caudal pole of the parotid gland. Initially, tumors present as an asymptomatic, slowly growing masses, affecting predominantly men (10:1) in the 5^th ^and 6^th ^decade [2,3]. Bilateral lesions occur in 7-10% of cases while multifocal lesions and recurrence occur in 2% of cases [4,5]. Histopathologically, they consist of a double-layer of oncocytic epithelial cells capable of forming cysts and papillary projections and a variable lymphatic stroma component [3]. Malignant transformation is very rare and the incidence of a carcinoma arising in a Warthin's tumor is approximately 1% [4]. Although pathogenesis is still unknown there is an association with cigarette smoking [3,6]. Since Honda et al. showed that the epithelial tumor components are polyclonal cell populations [7] these lesions cannot be classified as a true neoplasia anymore. This is supported by the fact that the tumors show a clinical behaviour that is typical for benign lesions and that there are only low rates for recurrence and malignant transformation [4,5,8]. Therefore, these lesions were classified as tumor-like-lesions [2,9].

There are several theories to explain the origin and the development of the tumors. Only two of the theories remain, namely the hypothesis that the tumor is an adenoma with concomitant lymphocytic infiltration [2], and second the hypothesis of heterotopia of salivary tissue into peri- and intraparotideal lymph nodes [10]. The latter theory is supported by the fact that during embryogenesis of the glandula parotis a penetration of epithelial cells of the oral mucosa into lymphocyte-rich tissue takes place. Due to the late encapsulation of the parotis the occurrence of intraparotideal lymph nodes and heterotopia of salivary tissue in parotideal and upper neck lymph nodes can be explained. Therefore, the origins of Warthin tumors are these epithelial inclusions [2,11,12]. This theory supports the heterotopia of salivary tissue in the upper neck and periparotideal region. It is unlikely to explain the origin of salivary tissue in the lower neck. Therefore, Youngs and Scofield suggested a relationship between a defective closure of the sinus of the His of the branchial apparatus with heterotopic salivary tissue [13].

There are only a few reports on the occurrence of extraglandular Whartin tumors, especially with a bilateral and multifocal appearance [11,14-16]. Here we present a rare case of a simultaneous bilateral, multifocal Warthin's tumor in upper cervical lymph nodes and give a brief review of the literature.

## Case report

A 85-year-old Caucasian man presented to our department in 2011 with a 4-year history of swelling on the mandibular angle of both sides. After initial slow and continuous growth, the masses now showed an accelerated enlargement in the last months, he indicated.

**Figure 1:**
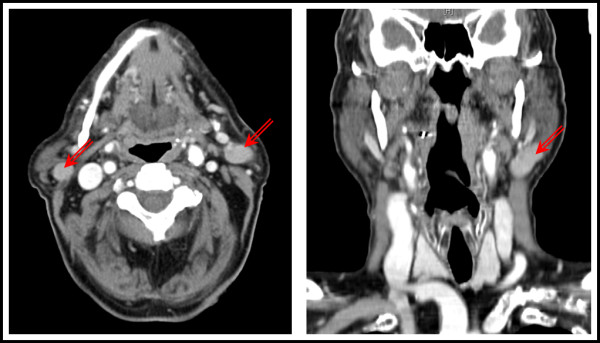
**Contrast-enhanced CT-scans showed a lesion next to the angle of the mandible on both sides (red arrows)**. The ovoid lesion showed a defined border with an intense uptake of the contrast medium.

In 2004 the patient had suffered from a carcinoma in situ of the right auricle that was treated by photodynamic therapy alio loco. At that time neither ultrasound nor a CT-scan showed any cervical masses. Follow-up care did not reveal any local recurrence of the carcinoma. In 2007, there had already been detected two enlarged lymph nodes of unknown dignity on the left (2.4 cm) and right (1.4 cm) angle of the mandible by ultrasound examination. The performed CT-scan classified the lymph nodes unspecific with a diameter of 1.6 cm on both angles of the mandible. During follow up, ultrasound was performed twice a year and showed no further enlargement of the lymph nodes. In the year 2011 ultrasound detected a mass on the left side of 3.5 cm and two masses of 1.5 and 1.6 cm on the right side, respectively. Due to their appearance they were classified as suspect lymph nodes. CT-scan paralleled these findings of suspect lymph nodes on both sides of the mandible (3.2 cm left, 1.8 cm right). The masses showed a defined border and an uptake of the contrast medium (Figure 1). No further masses or lymph nodes in the parotid glands or the neck were detected. Therefore the patient was submitted to our department in order to perform a lymph node biopsy.

The patient was a non-smoker. He reported no pain, facial or trigeminal disorders. B-symptoms, a stay abroad and the presence of domestic animals were denied. Furthermore, the patient suffered from coronary heart disease, cardiac arrhythmia, barrett esophagus and hypertension.

**Figure 2:**
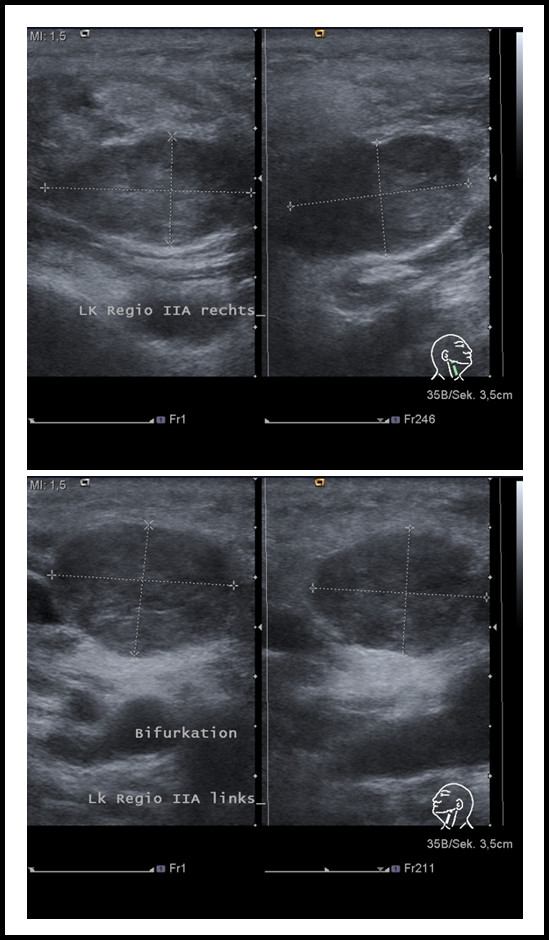
**Ultrasound examination of the lymph-nodes in regio IIA on both sides (upper panel: the lymph node on the right side; lower panel: the lymph node on the left side): The masses showed a widely defined border, a hypoechoic internal pattern with anechoic areas and a dorsal enhancement**. No vessels within the lesions could be detected by Doppler-ultrasound and the regular lymph node anatomy with hilum and cortex was depleted. Therefore, the lesions were suspicious of containing metastastic tumor masses.

Physical examination in our department showed soft, painless solid masses at the left and right angle of the mandible which were moveable to the skin and the deeper tissue. Ultrasound revealed two round-ovoid-shaped nearly fused lymph nodes in the left regio IIA cranial to the bifurcation of 16 and 15 mm in the maximum diameter, respectively. No vessels could be detected inside the lymph nodes, the regular lymph-node anatomy with hilum and cortex was absent. The internal echo was increased with interspersed hypoechoic areas. The borders of the lymph nodes were widely well-defined and they displayed a dorsal sound-enhancement. A very similar lymph node was found in the right regio IIA which measured 18 mm in the maximum diameter (Figure 2). Thus, to exclude metastatic tumor growth in the three lymph nodes we decided to excise them in toto without prior FNAC.

**Figure 3:**
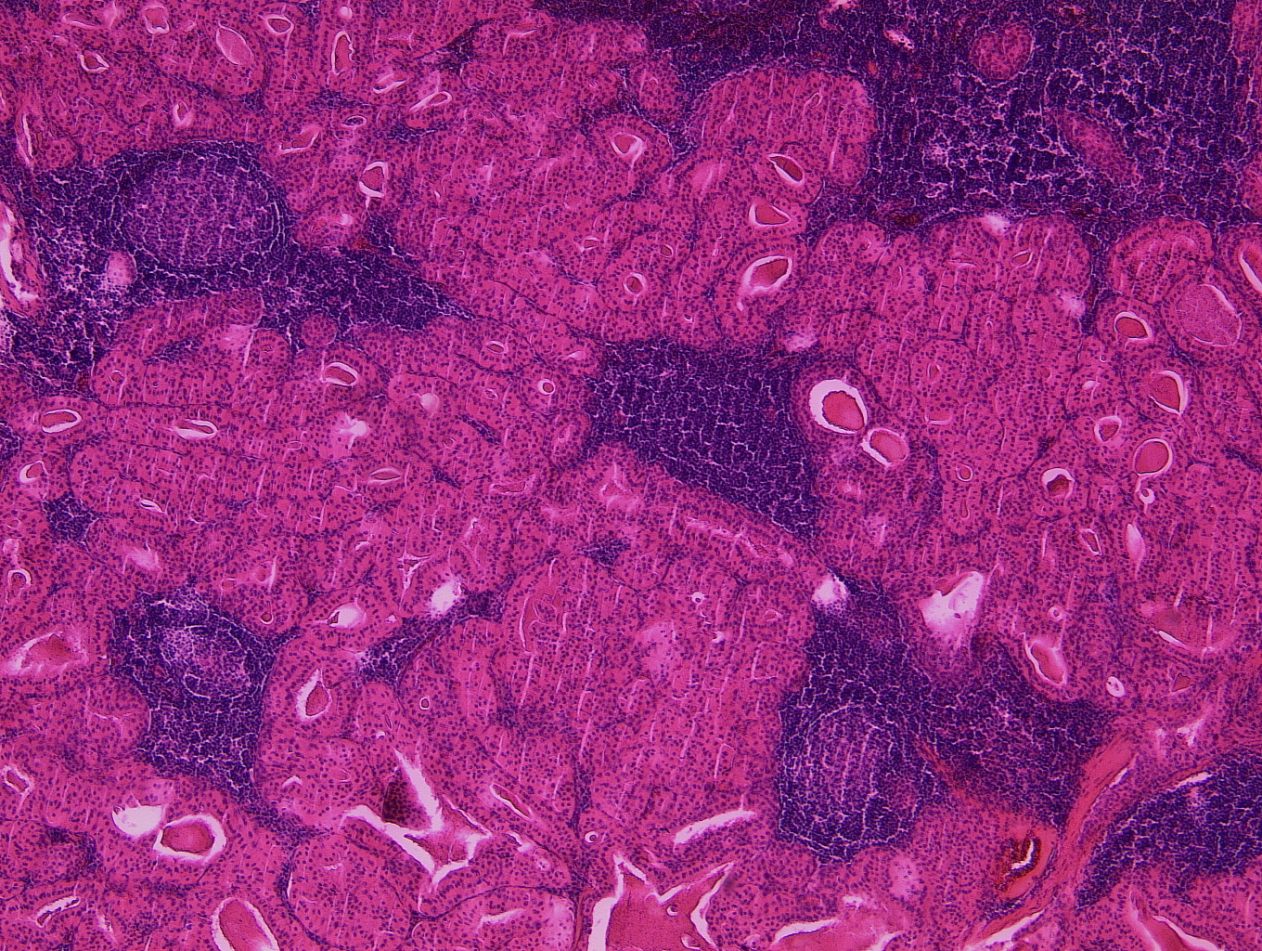
**HE-stained slide of one of the right-sided Warthin tumours**. Warthin tumour with the typical two-layered oncocytic epithelium forming closely packed tubules accompanied by a lymphoid stroma (50 × magnification).

Therefore, the patient underwent a selective lymph node excision on both sides in general anesthesia without any complications. Histological examination revealed oncocytic benign epithelial cells and mixed lymphoid cells consistent with Warthin's tumors in three cervical lymph nodes (Figure 3). On the left side there were two lymph nodes with a diameter of 1.5 cm each and on the right side the diameter of the mass was 3.2 cm.

The patient recovered from the operation very soon and showed no signs of recurrence during follow up so far.

## Discussion

The first description of adenolymphoma was done by Hildebrand in 1895 [17] and in 1929 Warthin published a series of so called papillary cystadenoma lymphomatosum leading to the well known term 'Warthin's tumor' [18]. Cystadenolymphomas are the second most frequent benign tumors of the parotid gland representing 6 - 10% of all tumors of the salivary glands [1]. However, it is difficult to estimate the real incidence of Warthin's tumors since there are regional, national and racial differences [2,3]. There is controversy about the origin and pathogenesis of cystadenolymphomas. One major hypothesis acts on the assumption of heterotopic salivary tissue. During the embryogenesis epithelial cells of the oral mucosa penetrate into lymphocyte-rich tissue and can be entrapped into parotideal lymph nodes. In addition, the late encapsulation of the parotid gland permits the occurrence of intraparotideal lymph nodes. Thus Warthin's tumors have their origin in these epithelial inclusions [2]. This hypothesis puts Warthin's tumors in vicinity of lateral cervical cysts, who are thought to origin from proliferating heterotopic inclusions of tonsillar tissue in cervical lymph nodes [3]. The hypothesis of heterotopia may explain the origin of salivary tissue in parotideal lymph nodes, in the periparotideal region and the upper neck, but fails to explain the origin of heterotopic salivary tissue in the lower neck or lymph nodes in the mediastinal cavity. Already in 1967 a relationship between a defective closure of the sinus of the His of the branchial apparatus with heterotopic salivary tissue was suggested by Youngs and Scofield [13] as a possible reason for heterotopic salivary tissue of the lower neck. Other authors propose that aberrant salivary tissue of the pharyngeal pouch may migrate downwards with the thyroid and parathyroid gland [19].

Beside the epithelial component these tumors consist of a stromal component with lymphocytic infiltrates and lymph follicles. One hypothesis about the origin of these lymphatic components is the proposal of Low et al., namely that the lymphatic component is a result of an immunological reaction to the epithelial growth [20]. This suggestion is supported by recent findings of Teymoortash et al. who could prove evidence for a promotion of lymphangiogenesis by the epithelial tumor [21].

Since Honda et al. were able to prove that the epithelial components are polyclonal cell populations this lesion cannot be classified as a true neoplasia anymore [7]. According to the WHO these lesions were classified as tumor-like-lesions [9]. There are many arguments that support the tumor-like classification of these lesions. First, these tumors show a slow growth and do not affect the facial nerve. This clinical appearance of this tumor is similar to other benign lesions. In addition, the polyclonal origin of the epithelial components excludes an malignant growth pattern [7]. In addition, there are low rates for recurrence and malignant transformation [4,5,8,22].

The sonographic diagnosis of neck masses originating from heterotopic salivary gland tissue remains challenging due to their rarity. In the present case, first of all, metastastic tumor growth had to be taken into account, because the whole lymphatic tissue was depleted of the lymph nodes. Moreover, the simultaneousness of the occurrence of three similar bilateral lesions suggested the presence of a metastasizing malignant tumor. The absence of detectable intra- or perinodal vessels and the hyperechoic appearance of the presumptive metastatic tissue rather argued against this assumption, but histopathological investigation of the entire lesions was the only way to establish the correct diagnosis.

The management of neck masses secondary to heterotopic salivary tissue is complex and depends among other things on the dignity of the lesion. Due to the fact that the definitive diagnosis can finally be achieved by a histological examination it is still a challenge for the surgeon to balance the surgery appropriate. Unnecessary surgery of the salivary glands with its associated morbidity or missing a primary malignancy should be avoided. Therefore a careful search for any primary cancer should be performed prior to the operation as recommended by Ferlito et al. [23]. In the presented case no primary malignancy could be detected by clinical examination, CT-scan and ultrasound. Afterwards the patient underwent a selective lymph node excision on both sides. This is consistent with the conclusion of Rodgers et al. namely that a local excision with close follow up is an appropriate management for benign neoplasms of cervical heterotopic salivary tissue [24]. According to Ferlito et al. a cervical lesion should be considered as primary tumor and treated as mentioned above if the preoperative search for primary malignancies is negative [23]. Consequently the exclusion of a major salivary gland neoplasm by clinical examination and radiographic imaging should preclude the removal of a major salivary gland [25]. There was a controversial discussion about the position of fine needle aspiration cytology in the diagnosis of Warthin's tumors or lesions of the parotid gland in general. Meanwhile several studies could prove a high sensitivity and specificity for the diagnosis of Warthin's tumor with fine needle aspiration cytology (FNAC) [26,27]. Risks like facial nerve lesions and infection can be disregarded because of their infrequency. Due to the fact that the final diagnosis cannot be established until histopathological study of the surgical sample has been performed, FNAC may be an option to close this gap of diagnosis and permit a detailed preoperative planning. In the presented case a FNAC was not performed because the masses were too far away from the parotid gland. Therefore a resection of the gland was beyond all questions. Nevertheless, a FNAC should be always performed for the preoperative diagnosis of lesions of the salivary glands if a resection of the gland is taken into account. Cytology, when coupled with clinical and image findings, may permit conservative tumor management [27].

## Conclusion

We presented a rare case of a multifocal bilateral cystadenolymphoma in upper cervical lymph nodes, the most frequent lesion of heterotopic salivary gland tissue. Even if there are several hypotheses the pathogenesis is still unclear. Recent studies were able to proof that these tumors are no true neoplasia and consequently were classified as tumor-like-lesions. The preoperative diagnosis to exclude primary malignancies and consequently the definition of a balanced surgery is still a major challenge for the surgeon. Careful clinical examination, radiography, ultrasound and in some cases fine-needle aspiration cytology should be performed. If a primary malignancy can be excluded the adequate therapy of these lesions is an isolated excision of the masses. In these cases surgery of the salivary glands with its morbidity should be avoided. Afterwards a close follow up is recommended.

## Consent

Written informed consent was obtained from the patient for publication of this Case report and any accompanying images. A copy of the written consent is available for review by the Editor-in-Chief of this journal.

## Competing interests

The authors declare that they have no competing interests.

## Authors' contributions

CN and CS wrote the paper, CS und DDS performed the operation, CS performed the ultrasound examination, SH did the histopathologic examinations, DDS collected the clinical data, RD, NRK and JH corrected and improved the paper. We confirm that no prior or duplicate publication or submission elsewhere of any part of the work has taken place. All authors have read and approved the final manuscript.
